# Influence of Temperature and Incidence Angle on the Irradiation Cascade Effect of 6H-SiC: Molecular Dynamics Simulations

**DOI:** 10.3390/mi14112126

**Published:** 2023-11-19

**Authors:** Yaolin Chen, Hongxia Liu, Cong Yan, Hao Wei

**Affiliations:** Key Laboratory for Wide Band Gap Semiconductor Materials and Devices of Education, School of Microelectronics, Xidian University, Xi’an 710071, China

**Keywords:** 6H-SiC, displacement damage, PKA, temperature, incidence angle

## Abstract

SiC devices have been typically subjected to extreme environments and complex stresses during operation, such as intense radiation and large diurnal amplitude differences on the lunar surface. Radiation displacement damage may lead to degradation or failure of the performance of semiconductor devices. In this paper, the effects of temperature and incidence angle on the irradiation cascade effect of 6H-SiC were investigated separately using the principles of molecular dynamics. Temperatures were set to 100 K, 150 K, 200 K, 250 K, 300 K, 350 K, 400 K and 450 K. The incidence direction was parallel to the specified crystal plane, with angles of 8°, 15°, 30°, 45°, 60° and 75° to the negative direction of the Z-axis. In this paper, the six types of defects were counted, and the microscopic distribution images and trajectories of each type of defect were extracted. The results show a linear relationship between the peak of the Frenkel pair and temperature. The recombination rate of Frenkel pairs depends on the local temperature and degree of aggregation at the center of the cascade collision. Increasing the angle of incidence first inhibits and then promotes the production of total defects and Frenkel pairs. The lowest number of total defects, Frenkel pairs and antisite defects are produced at a 45° incident angle. At an incidence angle of 75°, larger size hollow clusters and anti-clusters are more likely to appear in the 6H-SiC.

## 1. Introduction

SiC material is a typical third-generation semiconductor material. It has been used in space exploration, nuclear reactions and electrical equipment at high altitudes on plateaus. 3C-SiC is mainly used in photovoltaic applications [1]. 4H-SiC materials are mainly used to fabricate high-frequency and high-power devices [2]. 6H-SiC materials are mainly used for the manufacture of photoconductive detectors [3]. Researchers typically use Rutherford Backscattering Spectroscopy (RBS) and Transmission Electron Microscope (TEM) to probe the irradiation damage of SiC materials [4]. Wenhui Dong et al. studied different doses of Si, H ion injection experiments using Scanning Electron Microscopy (SEM) and Transmission Electron Microscopy. The results show that the irradiation damage effect of 6H-SiC is highly dependent on the type of irradiated ions, the amount of ion injection and the irradiation sequence [5,6]. W. J. Weber investigated defect clusters and amorphization induced by high-dose neutron irradiation in SiC materials [7]. The generation and recrystallization behavior of SiC amorphous crystals has been achieved by low-energy He and Fe ion injection [8,9]. C. Hemmingsson et al. conducted an investigation into the deep-level defects induced by electron irradiation in 4H-SiC using deep-level transient spectroscopy. They observed multiple electron traps and a single hole trap [10]. Concurrently, A. Castaldini et al. identified that among the five defect levels introduced by irradiation, three exhibited similar electrical characteristics [11]. Katoh et al. investigated the composite materials of SiC fibers and pyrocarbon under neutron irradiation conditions. They found that the density of defects significantly influences the post-neutron irradiation electrical properties of SiC semiconductors [12]. Wang Pengfei et al. found that the type and structure of irradiation-induced defects in 6H-SiC materials affect the extent of neutron irradiation damage. They observed that annealing temperature affects the recombination of defects and lattice recovery, ultimately influencing the resistivity of 6H-SiC materials [13]. Other researchers also found that the radiation damage caused by P ion injection partially recovered or even disappeared at different annealing temperatures and annealing times [14]. In addition, several researchers have investigated the displacement threshold energies and the laws of defect aggregation in SiC lattice atoms [15,16]. 

Silicon vacancies in silicon carbide (SiC) have been proposed as attractive candidates for quantum technology applications, such as quantum sensing and quantum repeaters [17]. As the use of 6H-SiC becomes more widespread, the complex environment poses new challenges for the application of 6H-SiC materials. Further understanding of the defect formation mechanism in 6H-SiC will help broaden the application fields of 6H-SiC. For example, in SiC device manufacturing technology, proton irradiation can be used to create localized regions of high resistance in a semiconductor [18]. Based on the principles of molecular dynamics (MD), this paper simulated the cascade effect of 6H-SiC at different temperatures and incidence directions. The evolution of point defects and clusters in 6H-SiC materials was studied under specific stress conditions.

## 2. Methods

**Figure 1:**
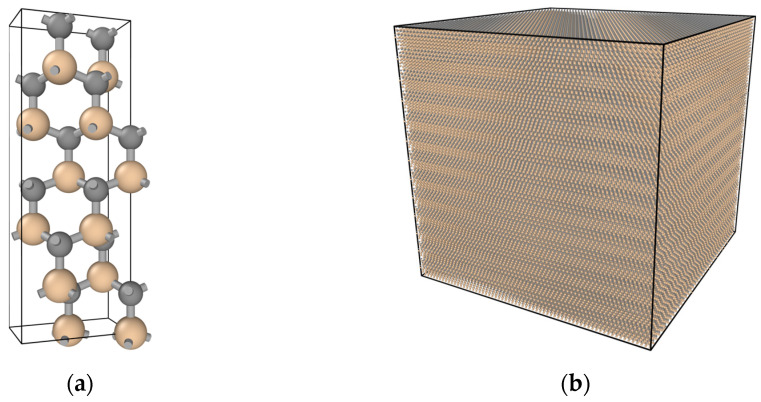
(**a**) Unit cell model for 6H-SiC; (**b**) Supercell model for 6H-SiC.

In this paper, irradiation experiments on 6H-SiC targets are modelled and simulated using the Large-scale Atomic\Molecular Massive Parallel Simulator (LAMMPS) [19]. 6H-SiC is a hexagonal crystal. The lattice constants are a = b = 3.08101 Å and c = 15.1248 Å. Table 1 shows the atomic coordinates of the unit cell of 6H-SiC [20]. Figure 1a,b show the unit cell and the supercell of the model for 6H-SiC, respectively. The supercell is obtained by expanding the unit cell.

**Figure 2:**
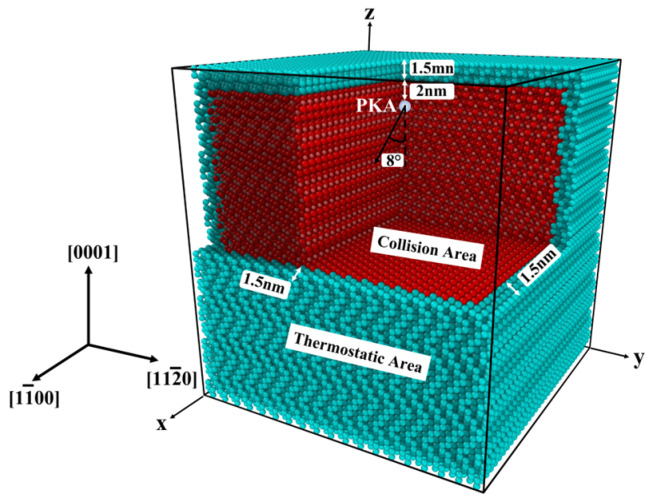
Schematic diagram of the irradiation model.

When low-energy particles irradiate materials, the main mode of energy loss is elastic collisions between atoms. Displacement cascades from cascade collisions are the main form of displacement damage. Figure 2 shows an irradiation model with fully periodic boundary conditions. Periodic boundary conditions ensure the integrity of the forces on the simulated system boundary atoms. The blue area in the model is a 1.5 nm thick constant temperature area. The temperature of the thermostatic area is controlled by the velocity rescaling (temp/rescale) method. The method is used to simulate the dissipation of energy. Collision area, the red area of the model, can be used to simulate the cascade collision effect of 6H-SiC. The Si-PKA is set in the inner part of the collision area, and the place is 2 nm from the thermostatic area on the upper surface. The aim is to avoid displaced atoms from cascade collisions entering the thermostat area. The incident direction of the PKA is parallel to the x-z plane and at an angle of 8° along the negative direction of the Z-axis.

The potential function describes the potential energy of the atoms in a material as a function of the positions of the atoms, i.e., it describes the forces between the atoms. The accuracy of the potential function parameters determines the accuracy of the simulation results. The potential function used in this paper is the Tersoff/ZBL potential function, whose parameters were confirmed in the experiments of Erhart et al. [21,22]. According to molecular dynamics, the atoms in the system obey the classical mechanics. The velocity of motion of each atom can be derived from the definition of velocity, i.e., the atoms in the system satisfy the set of Equations (1). As shown in Table 2, the simulation system was first relaxed by constant-pressure, constant-temperature ensemble (NPT) for 30 ps. And the micro-canonical ensemble (NVE) was used during the irradiation process. Table 3 shows the experimental design. Experiment 1 parameters are kept constant except temperature. Experiment 2 parameters are kept constant except for the angle of incidence.

(1) $$\left\{\begin{array}{c} F_{i}(t)=m_{i}\frac{\partial \nu_{i}(t)}{\partial t}\\ \nu_{i}=\frac{\partial r_{i}(t)}{\partial t} \end{array}\right..$$

$F_{i}(t)$ is the force on particle *i* at time t. $m_{i}$, $\nu_{i}$ and $r_{i}$ are the mass, velocity and coordinates of particle *i*, respectively.

**Figure 3:**
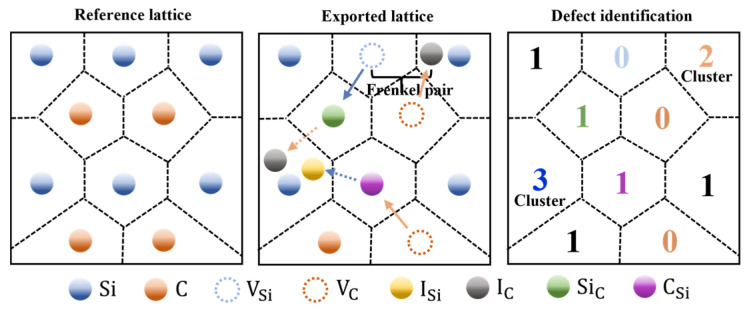
Schematic diagram of the Wiger–Seitz method.

Figure 3 is a schematic representation of the Wiger–Seitz method. The Open Visualisation Tool (OVITO) is used to identify and count the types and clusters of defects [23]. Frenkel pairs, silicon vacancy (V_Si_), carbon vacancy (V_C_), silicon interstitial (I_Si_), carbon interstitial (I_C_), silicon antisite (Si_C_), carbon antisite (C_Si_) and cluster can be shown in the OVITO. Among them, clusters have a search radius of 0.22 nm [16,24].

## 3. Results and Discussion

### 3.1. Validation of the Model

The density, radial distribution function, atomic angular distribution function and coordination number of the model are calculated in this section. In addition, to verify the reliability of the model, the experimental data from other researchers are compared in the following content.

**Figure 4:**
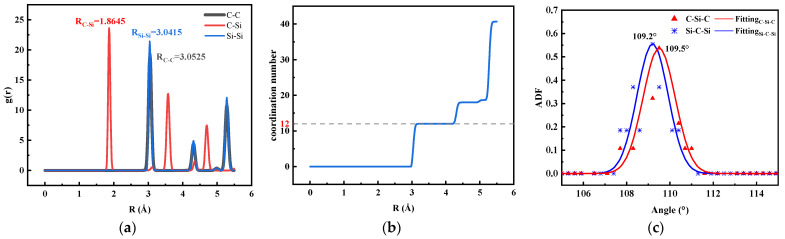
Structural data for the models in this paper. (**a**) Radial distribution function; (**b**) coordination number; (**c**) atomic angle distribution function.

Table 4 shows that the lattice constants after NPT optimization are not significantly different from the experimental values. Figure 4a shows that the bond length of C-Si is 1.8645 Å in the simulated system. Figure 4b shows that the coordination number of the 6H-SiC crystal is 12, consistent with the coordination number of the actual hexagonal crystal structure. Figure 4c shows angles of 109.5° and 109.2° for C-Si-C and Si-C-Si, respectively.

Table 5 shows the error statistics information. Compared with the experimental value [20], the density error of the model in this paper is about 3.302%. The error of the Si-C bond length is about −1.3388%. The atomic angle errors of Si-C-Si and C-Si-C are about −0.2439% and 0.0759%, respectively.

### 3.2. Effect of Temperature on 6H-SiC Cascade Collisions

In the field of space exploration, extreme environments pose a severe impact on equipment. For example, the temperature on the lunar surface can reach −183 °C or 127 °C. This subsection focuses on the variation in the 6H-SiC irradiation cascade effect at ambient temperatures of 100 K, 150 K, 200 K, 250 K, 300 K, 350 K, 400 K and 450 K.

**Figure 5:**
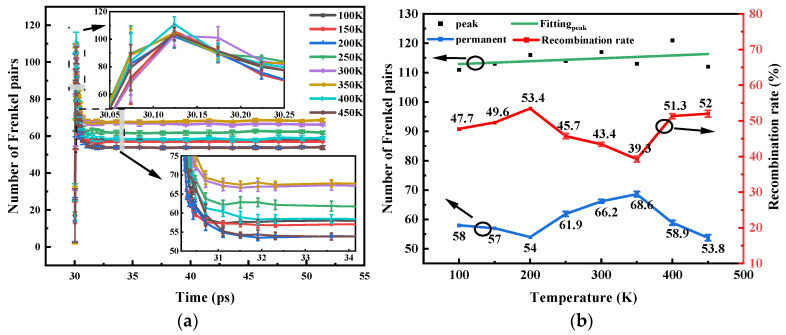
Production of a Frenkel pair at different temperatures. (**a**) Number of Frenkel pairs versus time; (**b**) Peak number of Frenkel pairs and permanent defects at different temperatures.

Figure 5a shows that different temperatures have no effect on the overall trend of defect evolution in 6H-SiC. The peak formation times of the Frenkel pairs are basically the same. The duration of cascade collisions becomes longer with increasing temperature. Figure 5b shows that the fitted curve between the peak of the Frenkel pair and the temperature satisfies a linear relationship, which can be expressed by Equation (2). The reason is that the transition from low to high temperatures enhances the thermal motion of the atoms. The energy of the lattice atoms is more likely to meet the displacement threshold energy. The permanent Frenkel pairs and their recombination rates have no obvious regularity. The paper further explains this phenomenon through the temperature nephogram.

(2) $$N_{max}=a+k*T,$$

where $N_{max}$ is the peak of the Frenkel pair, a is the intercept (a=111.94048±2.99957). k is the slope (k=0.00976±0.01007), and T is the temperature in Kelvin.

**Figure 6:**
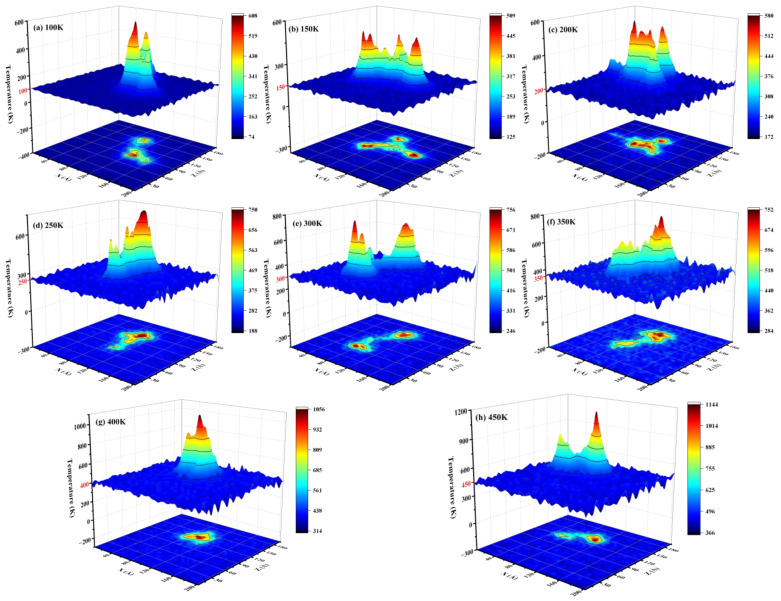
The nephogram at different temperatures. (**a**) 100 K; (**b**) 150 K; (**c**) 200 K; (**d**) 250 K; (**e**) 300 K; (**f**) 350 K; (**g**) 400 K; (**h**) 450 K.

Figure 6 shows the nephogram at different temperatures. A peak represents a cascade collision centre, corresponding to a recombination centre. As the temperature increases from 100 to 200 K, the number of recombination centres increases and the dispersion decreases. The result is an increase in the recombination rate of Frenkel pairs. As the temperature rises from 200 K to 350 K, the dispersion of the recombination centre increases, leading to a decrease in the recombination rate of Frenkel pairs. A larger cascade of atomic collisions occurs when the temperature is raised from 350 K to 450 K, corresponding to a local temperature of over 1000 K. Further, high temperature annealing promotes the recombination of defects [14].

**Figure 7:**
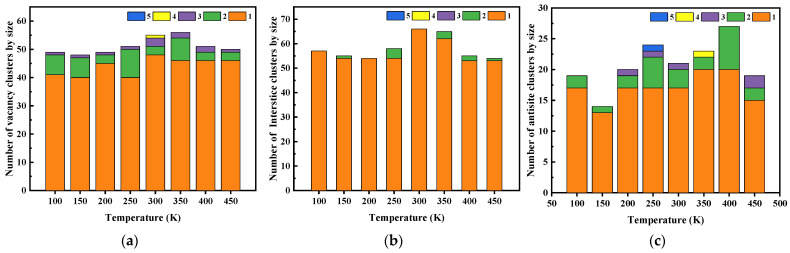
The number of clusters by size. (**a**) Vacancy clusters; (**b**) Interstice clusters; (**c**) Antisite clusters.

The barrier potential for various types of point defects in SiC is given in Table 6 [25,26,27]. Figure 7 shows that the irradiation defect types are mainly in the form of point defects at different temperatures. These clusters are mainly vacancy clusters and antisite clusters. The reason is that I_C_ and I_Si_ have relatively low migration potential. It is not easy to form interstitial clusters.

### 3.3. Effect of Incidence Angle on 6H-SiC Cascade Collisions

The incident angle of irradiated ions affects the particle deposition trajectory and hence the transient current of the device, a phenomenon that has been reported [28,29]. The 6H-SiC is an anisotropic crystal. Irradiated particles with different incidence angles may form different cascade collisions in 6H-SiC, leading to different levels of irradiation damage. Therefore, this section focuses on the effect of different incidence angles on 6H-SiC cascade collisions.

**Figure 8:**
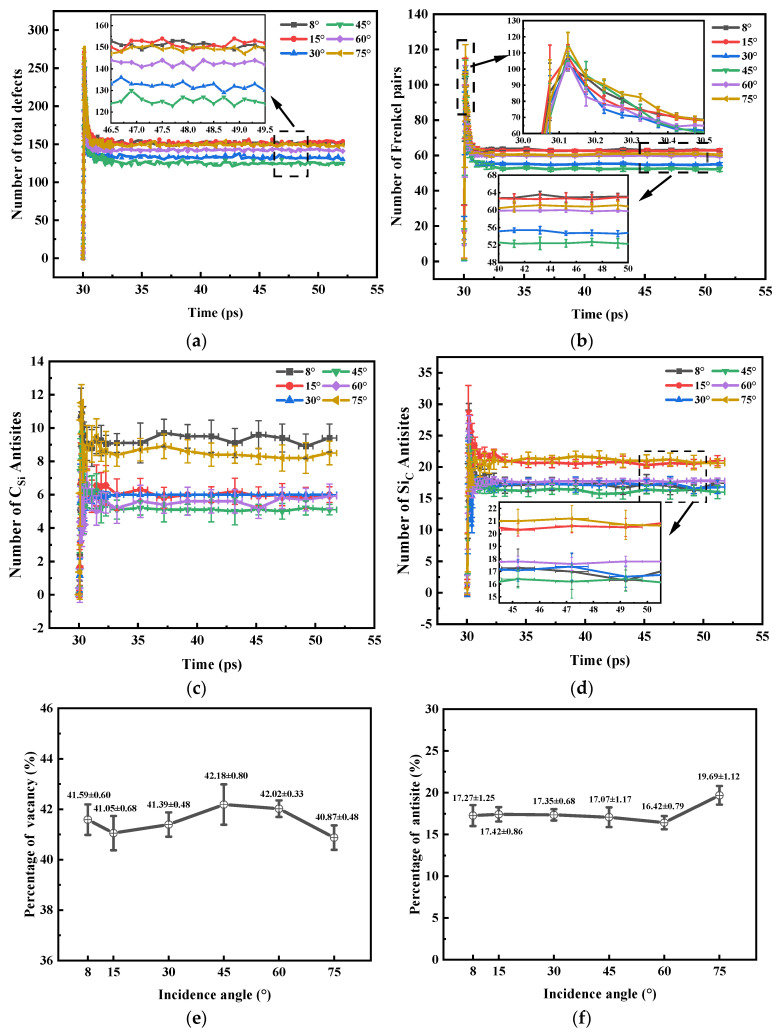
(**a**) Number of total defects versus time curves; (**b**) Number of Frenkel pairs versus time curves; (**c**) Number of C_Si_ versus time curves; (**d**) Number of Si_C_ versus time curves; (**e**) Percentage of vacancy; (**f**) Percentage of antisite.

Figure 8a shows that the number of permanent defects produced by cascade collisions tends to decrease and then increase as the angle of incidence increases. When the incidence angles are 8° and 15°, the total number of defects is roughly the same. The number of total defects, Si_C_ and C_Si_, are the least at the 45° incident angle. Among them, the total defect consists of V_C_, V_Si_, I_C_, I_Si_, Si_C_ and C_Si_. Figure 8b shows a similar regularity for the number of permanent Frenkel pairs generated by cascade collisions. Figure 8c indicates that the number of permanent C_Si_ tends to decrease and then increase as the angle of incidence increases. When the incidence angle is 15° and 30°, the difference in C_Si_ quantity is very small. Figure 8d shows that there is no obvious regularity in the number of Si_C._ To further explain the differences in the number of defects in each category, we provide reactions between the various types of point defects studied and their reaction barrier (E_Re_) and reaction distances (r) [27,30]. In Table 7, the reaction barriers for C_C_ and Si_Si_ are smaller than those of the other forms. Therefore, during the defective recombination phase, a large number of recombination occurs between V_C_ and I_C_ and between V_Si_ and I_Si_. This leads to a large reduction in Frenkel pairs as shown in Figure 8b. However, by comparison with Figure 8b,c, it can be observed that the amount of C_Si_ does not change significantly during the defect recombination phase. The recombination of Si atoms in Si_C_ and V_Si_ is the reason for the decrease in the amount of Si_C_ in Figure 8d. This also suggests that the Si_C_ produced at the beginning of irradiation is not stable Si_C_, but a combination of V_C_ and I_Si_. As shown in Table 8 [26,27], the formation energy of Si_C_ is lower than that of C_Si_, so the peak defects in Figure 8c are lower than those in Figure 8d. The results in Figure 8e,f show that the variation in the incidence angle has no significant effect on the final percentage of vacancy defects and antisite defects. This shows that the effect of the variation in the PKA incidence angle on the electrical properties of the electronic devices is more in the regional distribution of defect clusters of vacancy defects and interstitial atomic defects generated within the material.

**Figure 9:**
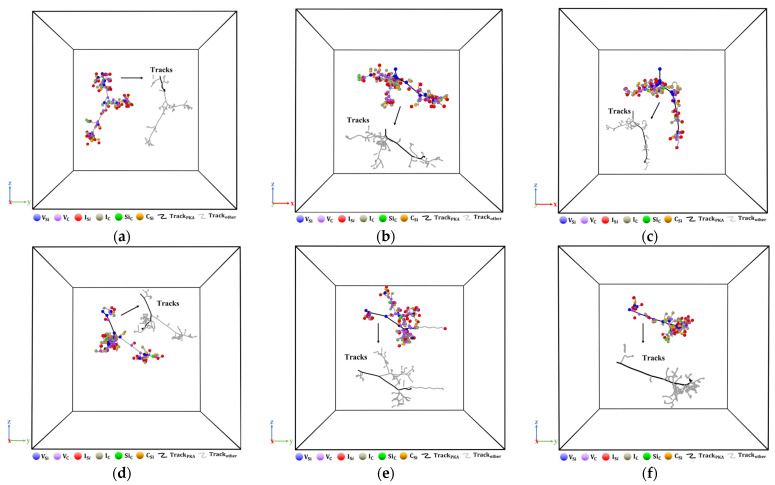
Microscopic distribution of point defects. (**a**) 8°; (**b**) 15°; (**c**) 30°; (**d**) 45°; (**e**) 60°; (**f**) 75°.

Figure 9 shows the microscopic distribution of various defect types. The trajectories in the figure are produced by atoms with displacements greater than 4 Å. Defects are distributed in the shape of grape bunches near the PKA trajectory. As the angle of incidence increases, local cascade collisions become more intense, and the aggregation of defects becomes more pronounced at the end of the PKA trajectory. Consistent with the above results, the local amorphization is more obvious within the 6H-SiC material. A distinct region characterized by the aggregation of vacancy defects and interstitial atomic defects emerges irrespective of alterations in the incident angle. Furthermore, this region undergoes modifications as the incident angle varies.

**Figure 10:**
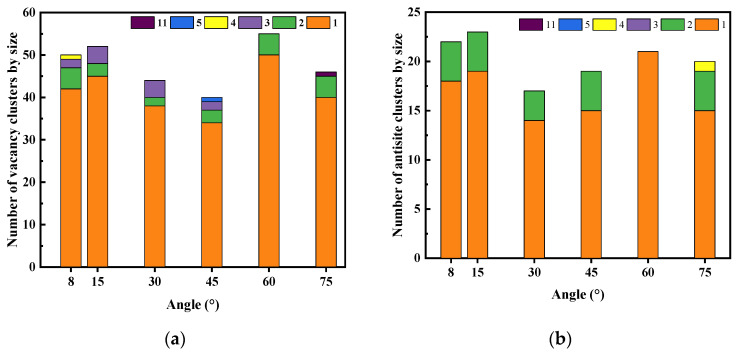
The number of clusters by size. (**a**) Vacancy clusters; (**b**) Antisite clusters.

Figure 10 shows that vacancy clusters and antisite clusters with an atomic number of two are the most common. The largest size of vacancy clusters and antisite clusters appear at a 75° incident angle. At an incident angle of 60°, there are no antisite clusters and no vacancy clusters greater than two. However, it can be concluded from Table 6 that the migration potentials of vacancy and antisite defects are much higher than those of interstitial defects, and therefore the clusters formed are mainly vacancy and antisite clusters.

## 4. Conclusions

During irradiation of 6H-SiC crystals, there is a monotonically increasing linear relationship between the peak of the Frenkel pair and temperature. The peak of the Frenkel pair increases with increasing temperature, but the amount of change is small. The recombination rate of Frenkel pairs depends on the local temperature and the degree of defective recombination center aggregation. As the temperature increases from 300 K to 450 K, the cascade collision distribution tends to concentrate, and local high temperatures are more likely to occur. As the angle of incidence increases, the number of both total defects and Frenkel pairs exhibits a trend of first decreases and then increases. Among them, the number of defects is minimal at a 45° incident angle. At a 75° incident angle, larger vacancy clusters and antisite clusters are produced. As the angle of incidence increases, secondary trajectories from irradiation cascade effect converge towards the end of the PKA trajectory.

## Figures and Tables

**Table 1:** Atomic coordinates for the 6H-SiC moissanite [15].

Atom	*x*	*y*	*z*
Si 1	0	0	0
Si 2	0.3333	0.6667	0.1664
Si 3	0.6667	0.3333	0.3329
Si 4	0.3333	0.6667	0.0412
Si 5	0.6667	0.3333	0.2080
Si 6	0	0	0.3746

**Table 2:** Parameters related to the time step.

Stage	Time Step (fs)	Number of MD Steps	Time (ps)
Relaxation phase (NPT)	1	30,000	30
Cascade collision phase (NVE)	0.01	50,000	0.5
Defect compounding stage (NVE)	0.1	20,000	2
Steady-state phase (NVE)	1	20,000	20

**Table 3:** The design of the experiment.

Experimental Group ^1^	Temperature (K)	Angle of Incidence (°)	Velocity Component of the y-Axis ^2^ (Å/ps)	Velocity Component of the z-Axis (Å/ps)
Experiment 1: Different ambient temperatures	100, 150, 200, 250, 300, 350, 400, 450	8°	257.8014	−1834.3527
Experiment 2: Different angles of incidence	300	8°	257.8014	−1834.3527
	300	15°	479.4312	−1789.2616
	300	30°	926.18997	−1604.2081
	300	45°	1309.8304	−1309.8304
	300	60°	1604.2081	−926.1900
	300	75°	1789.2616	−479.4312

^1^ The dimensions of the model are 65a × 38b × 13c. Total atomic number is 770,640. ^2^ The velocity component of the *x*-axis is 0.

**Table 4:** Lattice constants.

Lattice Constants ^1^ (Å)	This Work (before NPT)	This Work (after NPT)	Reference [15]
a	3.0810	3.080999	3.0810
b	5.3364	5.336400	5.3364
c	15.1248	15.124799	15.1248

^1^ The hexagonal lattice constants in the table were all converted to orthogonal lattice constants.

**Table 5:** Error statistics table.

	This Work ^1^	Reference [15]	Average Error ^2^ (%)
Density (g/mol)	3.316	3.21	3.302
Bond lengths (Å)	1.8645	1.8898	−1.3388
Angles (°)	109.2 (Si-C-Si)	109.467 (Si-C-Si)	−0.2439
	109.5 (C-Si-C)	109.417 (C-Si-C)	0.0759

^1^ The temperature is 300 K. ^2^ ± indicates that the value in this paper is greater or less than the experimental value.

**Table 6:** Potential barriers for various types of point defects.

	V_C_	V_Si_	I_C_	I_Si_	Si_C_	C_Si_
Migration potential/eV	3.66	3.20	0.67	1.48	11.60	11.70

**Table 7:** The recombination pattern of point defects, reaction barriers (E_Re_) and reaction distances (r) [27,30].

Recombination Pattern	Reaction Barriers (E_Re_)/eV	Reaction Distances (r)/Å
I_C_ + V_C_ → C_C_	0.43	3.08
I_Si_ + V_Si_ → Si_Si_	0.17	5.34
I_C_ + V_Si_ → C_Si_	1.25	3.30
I_Si_ + V_C_ → Si_C_	1.11	3.70
I_C_ + Si_C_ → C_C_ + I_Si_	1.34	4.36
I_Si_ + C_Si_ → Si_Si_ + I_C_	0.64	4.36

**Table 8:** The formation energy of defects [26,27].

	V_C_	V_Si_	I_C_	I_Si_	Si_C_	C_Si_
Formation energy/eV	4.19	4.97	6.95	8.75	3.56	4.03

## Data Availability

Data are contained within the article.
